# Abnormal dynamic functional connectivity changes correlated with non-motor symptoms of Parkinson’s disease

**DOI:** 10.3389/fnins.2023.1116111

**Published:** 2023-03-16

**Authors:** Yuanyan Cao, Qian Si, Renjie Tong, Xu Zhang, Chunlin Li, Shanhong Mao

**Affiliations:** ^1^School of Biomedical Engineering, Capital Medical University, Beijing, China; ^2^Beijing Advanced Innovation Center for Big Data-Based Precision Medicine, Beijing, China; ^3^Beijing Key Laboratory of Fundamental Research on Biomechanics in Clinical Application, Capital Medical University, Beijing, China; ^4^School of Cyber Science and Technology, Beihang University, Beijing, China

**Keywords:** Parkinson’s disease, dynamic functional connectivity, independent component analysis (ICA), static functional connectivity, non-motor symptoms

## Abstract

**Background:**

Non-motor symptoms are common in Parkinson’s disease (PD) patients, decreasing quality of life and having no specific treatments. This research investigates dynamic functional connectivity (FC) changes during PD duration and its correlations with non-motor symptoms.

**Methods:**

Twenty PD patients and 19 healthy controls (HC) from PPMI dataset were collected and used in this study. Independent component analysis (ICA) was performed to select significant components from the entire brain. Components were grouped into seven resting-state intrinsic networks. Static and dynamic FC changes during resting-state functional magnetic resonance imaging (fMRI) were calculated based on selected components and resting state networks (RSN).

**Results:**

Static FC analysis results showed that there was no difference between PD-baseline (PD-BL) and HC group. Network averaged connection between frontoparietal network and sensorimotor network (SMN) of PD-follow up (PD-FU) was lower than PD-BL. Dynamic FC analysis results suggested four distinct states, and each state’s temporal characteristics, such as fractional windows and mean dwell time, were calculated. The state 2 of our study showed positive coupling within and between SMN and visual network, while the state 3 showed hypo-coupling through all RSN. The fractional windows and mean dwell time of PD-FU state 2 (positive coupling state) were statistically lower than PD-BL. Fractional windows and mean dwell time of PD-FU state 3 (hypo-coupling state) were statistically higher than PD-BL. Outcome scales in Parkinson’s disease–autonomic dysfunction scores of PD-FU positively correlated with mean dwell time of state 3 of PD-FU.

**Conclusion:**

Overall, our finding indicated that PD-FU patients spent more time in hypo-coupling state than PD-BL. The increase of hypo-coupling state and decrease of positive coupling state might correlate with the worsening of non-motor symptoms in PD patients. Dynamic FC analysis of resting-state fMRI can be used as monitoring tool for PD progression.

## 1. Introduction

There were 6.1 million Parkinson’s disease (PD) patients worldwide in 2016 (GBD 2016 Neurology Collaborators, 2018). PD progresses continuously over time, mainly affecting older people (Poewe et al., 2017). Motor symptoms, such as tremors, akinesia, and rigidity, are the main symptoms of PD, impacting a patient’s ability to perform routine tasks and increasing the health care burden (GBD 2016 Neurology Collaborators, 2018). The occurrence of non-motor symptoms, such as cognitive impairment, depression, autonomic dysfunction and sleep disorders, progresses over time, resulting in deteriorating quality of life (Khoo et al., 2013; Diederich et al., 2020; Bloem et al., 2021). Occurrence of non-motor symptoms may earlier than motor symptoms, even in prodromal stage. Non-motor symptoms of PD may be used as early diagnosing biomarkers. Dopamine supplements and dopamine transporter agonists are commonly used treatments for PD that relieve motor symptoms. Treatments for PD non-motor symptoms were similar to those used for the general population (Armstrong and Okun, 2020; Vijiaratnam et al., 2021).

There are many ways to explore PD mechanisms. Functional magnetic resonance imaging (fMRI) is a non-invasive method to reveal brain activity by detecting blood oxygen level-dependent (BOLD) signals (Wu and Hallett, 2005; Smith et al., 2013a,b; Barkhof et al., 2014). The functional connectivity (FC) method measures correlations between time courses of regions of interest (ROIs) using Pearson correlation analysis (Shahhosseini and Miranda, 2022). FC analysis revealed that intrinsic networks existed in the brain (Allen et al., 2014) and was also used to explore the PD mechanism (Schindlbeck and Eidelberg, 2018; Ryman and Poston, 2020). FC within the motor network was correlated with the severity of PD motor symptoms (Chung et al., 2020). FC between the left amygdala and thalamus was increased in PD patients with depression compared to PD patients without depression (Hu et al., 2015). Anxiety severity of PD was positively correlated with their FC between the amygdala and superior parietal lobule (Zhang et al., 2019).

Unlike static FC analysis, dynamic FC analysis focuses on FC variability over time (Calhoun et al., 2014). Dynamic FC analysis was more sensitive and revealed more detailed information than static FC analysis (Du et al., 2017). The sliding window method is the most commonly used method for dynamic FC analysis, and a 30–60 s window size is appropriate, according to Allen et al. (2014). The FC matrix of each window can be grouped into different clusters, also known as states. Changes in temporal characteristics revealed PD mechanisms (Kim et al., 2017), and PD subjects with rapid eye movement (REM) sleep behavior disorder (RBD) spent more time in the state characterized by weaker positive coupling between the visual and default networks, and default and basal ganglia networks (Gan et al., 2021). PD patients with dementia spend increased time in a state with positive coupling within networks (Fiorenzato et al., 2019), while PD patients with mild cognitive impairment showed decreased connectivity between networks, especially between the sensorimotor and cognitive control networks (Díez-Cirarda et al., 2018). Compared with seed-based analysis, independent component analysis (ICA) reduces noise signals (Calhoun et al., 2001). Voxels in independent components were characterized by similar time courses and spatial distributions (Esposito et al., 2002).

Progressive dynamic FC changes during PD duration have rarely been studied. Non-motor symptoms of PD may occur before motor symptoms, and have potential to be early diagnosing biomarker. Non-motor symptoms exist through whole disease duration and become severer, which lead the potential to be progression biomarker of PD (Tolosa et al., 2021). So, it is important to explore the mechanisms of non-motor symptoms. fMRI method focused on fluctuations in the blood oxygen level dependent (BOLD) signal of different brain areas (Lee et al., 2013). fMRI was widely used in neuroscience studies (Smith et al., 2013a,b; Raimondo et al., 2021) and provided insight into the mechanism and diagnosis of PD (Tessitore et al., 2019; Wolters et al., 2019; De Micco et al., 2021).

Static FC and dynamic functional signal changes reflected brain activities. Correlations between functional changes in the brain and non-motor dysfunctions remain unclear. We hypothesize that the progression of non-motor symptoms may be associated with static FC changes and temporal characteristic dynamic FC changes. We utilized long term data of patients from PPMI dataset to address above questions. Static and dynamic FC between and within intrinsic networks were analyzed to determine static and dynamic FC changes during disease duration and correlations between FC changes and non-motor dysfunctions.

## 2. Materials and methods

### 2.1. Participants

Data used in the preparation of this article were obtained from the Parkinson’s Progression Markers Initiative (PPMI) database,^1^ which is a multicenter international study. For currently updated information on this study visit.^2^ All data used in our study were downloaded before January 2021. For detailed inclusion criteria of the PPMI dataset, please visit (see text footnote 2).

A total of 101 PD patients scanned fMRI were recruited in PPMI dataset. We selected patients with below inclusion criteria to our study: (1) visited for at least 4 years; (2) scanned fMRI at baseline (PD-BL) and followed-up (PD-FU); (3) older than 50 years old, as pathogenesis of late-onset PD and early onset PD is different (Schrag and Schott, 2006; Fereshtehnejad and Posteuma, 2017) and this study focused on late-onset PD. We focused on the late-onset PD, because late-onset PD population was larger than early onset PD patients. Late- and early onset PD were separated in many studies (Rango et al., 2021; Sigirli et al., 2021). The patients with low image quality or diagnosed with other diseases, such as Alzheimer’s disease, multiple system atrophy, dementia with Lewy bodies, during visiting period were excluded from this study. Imaging data and information of Healthy control (HC) subjects were also downloaded from PPMI dataset, who were scanned fMRI. In total, data of 20 PD patients and 19 healthy control subjects were utilized in our study.

### 2.2. Neuropsychological and clinical assessments

The disease severity of PD patients was assessed using the Movement Disorder Society Unified Parkinson Disease Rating Scale (MDS-UPDRS) (Goetz et al., 2008), Part I of the UPDRS was used to evaluate non-motor symptoms of PD, and parts II and III evaluated motor symptoms. Total UPDRS was sum of part I-IV. Hoehn and Yahr (H and Y) staging (Hoehn and Yahr, 2001) were used to assess severity of PD. The UPDRS-III and H and Y staging scores used in this study were assessed at “ON” state, which was defined as the last dose of levodopa or dopamine agonist were taken <6 h before assessment. The “OFF” state meant the last dose of levodopa or dopamine agonist were taken ≥6 h before assessment. The drug treatment dosage was calculated as the levodopa-equivalent daily dose (LEDD) (Tomlinson et al., 2010). Patients’ cognitive states were assessed using the Montreal Cognitive Assessment (MoCA) scale, Benton Judgment of Line Orientation Score (BJLOT), Letter Number Sequencing Score (LNS), Semantic Fluency Total Score (SFT), Symbol Digit Modalities Score (SDM), and Hopkins Verbal Learning Test–Revised (HVLT) scales. The total SFT score and subtest such as animal, fruit and vegetable scores were calculated in this study. HVLT contains several aspects, such as discrimination scores, immediate/recall scores, retention score, false alarms, delayed recall and delayed recognition. The ability of daily living (ADL) scale was used to evaluate life quality. Depression and anxiety were assessed by the Geriatric Depression Scale (GDS) score and State-Trait Anxiety Inventory (STAI). The University of Pennsylvania Smell Identification Test (UPSIT) was used to evaluate olfactory nerve dysfunction. Sleepiness quality and rapid eye movement (REM) sleep were assessed by the Epworth Sleepiness Scale (ESS) score and REM Sleep Behavior Disorder Questionnaire score. Autonomic function was assessed by the Scales for Outcomes in Parkinson’s Disease-Autonomic dysfunction (SCOPA-AUT).

### 2.3. MRI acquisition

T1 MRI and rs-fMRI data were collected on Prisma fit 3-tesla Siemens MR scanner (Siemens, Erlangen, Germany). All participants were requested to remain calm and not think during scanning. T1 MRI data were acquired with the following parameters: repetition time (TR) = 2,300 ms, echo time = 3.0 ms, flip angle = 9°, matrix size = 176 × 240 × 170, and voxel size = 1 mm × 1 mm × 1 mm. The rs-fMRI data, consisting of 210 volumes per patient, were acquired with TR = 2.4 s, echo time = 25 ms, flip angle = 80°, matrix size = 68 × 66 × 40, and voxel size = 3.3 mm × 3.3 mm × 3 mm.

### 2.4. MRI data processing

The preprocessing of the fMRI data was conducted using the functional connectivity (CONN) toolbox (Whitfield-Gabrieli and Nieto-Castanon, 2012),^3^ based on MATLAB (R2018a, MathWorks, Inc., Natick, MA, USA). The first ten scans were removed to maintain the stability of fMRI data. Thus, each participant provided 200 volumes for further analysis.

A slice-timing correction step was performed to correct the image acquisition time between slices. The fMRI data were realigned to the first volume to correct for head movement and then co-registered to the T1 MRI data using an affine transformation. The T1 MRI data were normalized to standard Montreal Neurological Institute (MNI) space and segmented into gray matter, white matter, and cerebrospinal fluid using the tissue probability maps. A non-linear transformation was applied to the functional data with the same parameters as the T1 MRI data. The functional data were smoothed with a Gaussian kernel of 6 mm full width half maximum, to reduce the noise signal.

### 2.5. ICA analysis

Independent component analysis (ICA) grouping was performed to obtain specific data components using Group ICA of the functional MRI Toolbox (GIFT v4.0 c).^4^ Voxel-level variance normalization was performed on all data. The number of independent components was estimated using a minimum description length approach. Functional data were decomposed using two data reduction steps (subject-specific and group-level PCA). The Infomax method was used to calculate independent components, and the ICASSO method implemented in GIFT was used to maintain reliability.

ICASSO was run 100 times, and 80% similarity was selected. Significant components from all independent components were chosen according to the location of the peak coordinate and time course characteristics (Allen et al., 2014; Kim et al., 2017). The selected components were identified as seven resting state networks (RSN): the default modal network (DMN), attention network (ATT), basal ganglia network (BG), visual network (VIS), sensorimotor network (SMN), auditory network (AUD), and frontoparietal network (FP) (Yeo et al., 2011).

### 2.6. Static functional connectivity analysis

The time courses of the selected components were extracted. Correlations between every pair of selected components formed the subject-specific static FC matrix. Static FC group comparisons were calculated among HC, PD-BL, and PD-FU using the multivariate analysis of covariance (MANCOVA) toolbox contained in GIFT with a corrected false discovery rate (FDR). *Post hoc* analyses were performed between HC and PD-BL and between PD-BL and PD-FU.

### 2.7. Dynamic functional connectivity analysis

Dynamic FC analysis was conducted using the dynamic functional network connectivity toolbox (dFNC) contained in the GIFT toolbox. The time courses of selected components were linear detrended and 3D-despiked. The time courses were also filtered using a low-pass filter with a cutoff of 0.15 Hz, as recommended in the GIFT manual.

A sliding window approach was used to analyze dynamic functional connectivity changes. The sliding window method is the most commonly used method for dynamic FC analysis, and a 30-60 s window size is appropriate, according to previous studies (Allen et al., 2014; Kim et al., 2017). The sliding window size was set to 22 TR (52.8 s) and the step was set to 1 TR (2.4 s), based on previous research (Allen et al., 2014; Kim et al., 2017). As a result, each participant had 178 windows across the entire scan. Correlations between the time course of each selected component pair in each window formed dynamic FC matrices. The K-means clustering method was performed to sort all dynamic FC matrices into different clusters, also known as states. A penalty on the L1 norm was imposed with 100 repetitions, and the elbow method was used to decide the clustering number.

Temporal properties of the dynamic FC analysis, such as fractional windows, mean dwell times, and numbers of transitions, were calculated for further statistical analysis. The fractional window is the proportion of time spent in each state, and the mean dwell time is the average time spent by each participant in each state. The number of transitions is calculated as the number of times switching between states.

### 2.8. Statistical analysis

Group differences in general information between the HC and PD groups, such as age, gender, years of education, and clinical assessments, were calculated using the two-sample *t*-test and the chi-square test. Differences in temporal properties among HC and PD subgroups were calculated using two sample *t*-test or rank sum test. Statistical differences of clinical assessments and temporal properties between PD-BL and PD-FU were tested using paired *t*-test or Wilcoxon signed rank test. Selecting *t*-test or non-parametric test was depended on the result of normality test. Correlations between temporal properties and clinical assessment scores were analyzed using partial correlation analysis, which controlled effect of age, sex, and education years. Above statistical analysis was performed in SPSS Statistics 22.0 (IBM Corporation, Armonk, NY, USA), and the threshold for statistical significance was *p* < 0.05. Multiple comparisons of *p*-values were corrected with FDR correction.

## 3. Results

### 3.1. Demographics

Twenty PD patients (13 male and 7 female) and 19 healthy subjects (15 male and 4 female) met the inclusion and exclusion criteria described in (Section “2.1. Participants”). There were no significant differences in age and gender between the PD and HC groups. The years of education of the PD group were statistically shorter than those of the HC group. After years of follow-up, the H and Y stage (ON state) of the PD group increased. The LEDD of PD-FU was significantly higher than PD-BL. These results indicated that the severity of PD increased after years of follow-up. The UPDRS-I scores of PD-FU increased compared to PD-BL. The HVLT-False alarms and SDM scores of PD-FU were smaller than HC group. The SCOPA-AUT scores of PD-FU were bigger than HC group. The UPSIT scores of PD-BL were smaller than HC group. Above results indicated that the non-motor symptoms of PD patients were worsen after years of follow-up. Other clinical assessments, such as UPDRS-III (ON state), total UPDRS, MoCA, BJLOT, ESS, GDS, SFT, and STAI scores, did not significantly change between PD-BL and PD-FU. Details of these results are shown in Table 1.

**TABLE 1:** Demographic information for the HC and PD subgroups.

				BL-HC		FU-BL		FU-HC	
	PD-BL (*n* = 20)	PD-Y3 (*n* = 20)	HC (*n* = 19)	*P*-value	*T/Z* value	*P*-value	*T* value	*P*-value	*T* value
Age (years)	64.35 ± 7.96	67.35 ± 8.0	62.84 ± 10.5	0.62	0.51	−	−	−	−
Gender (M: F)	13: 7	–	15: 4	0.33	0.94	−	−	−	−
Education years	13.4 ± 3.2	–	16.84 ± 2.54	**0**.**002**	-3.16	−	−	−	−
Disease duration (months)	4.99 ± 4.82	–	–	−	−	−	−	−	−
LEDD	215.65 ± 164.61	590.36 ± 229.67	–	−	−	**<0**.**001**	5.98	−	−
H and Y stage (ON)	1.37 ± 0.48	1.7 ± 0.47	–	−	−	**0**.**03**	2.18	−	−
ADL	90 ± 6.88	89.25 ± 5.68	–	–	−	0.51	-0.66	–	−
UPDRS-I	6.7 ± 5.17	8.15 ± 5.03	–	−	−	**0**.**03**	2.42	−	−
UPDRS-II (ON)	6.4 ± 4.59	7.65 ± 4.73	–	−	−	0.46	0.74	−	−
UPDRS-III (ON)	15.9 ± 9.83	18.1 ± 9.22	–	−	−	0.35	0.94	−	−
Total UPDRS (ON)	29.26 ± 15.38	33.9 ± 15.15	–	−	−	0.23	1.25	−	−
HVLT-immediate recall	26.05 ± 6.12	25.8 ± 5.91	25.89 ± 5.08	0.70	0.38	0.83	0.21	0.87	0.17
HVLT-delayed recall	9.1 ± 2.94	8.4 ± 3.42	8.21 ± 3.12	0.38	0.88	0.26	-1.13	0.91	0.11
HVLT-recognition	10.8 ± 2.73	11.2 ± 1.11	11.21 ± 1.03	0.68	0.42	0.85	-0.21	0.84	0.20
HVLT-false alarms	0.8 ± 0.95	0.6 ± 0.75	2.16 ± 2.17	0.06	-1.91	0.34	-1.10	**0**.**02**	-2.33
HVLT-discrimination	10 ± 2.92	10.6 ± 1.6	7.16 ± 5.52	0.11	1.58	0.76	-0.36	0.05	1.92
HVLT-retention	0.84 ± 0.2	0.8 ± 0.24	0.79 ± 0.23	0.55	0.60	0.38	-0.88	0.89	0.14
BJLOT	12.6 ± 1.93	13.3 ± 1.56	13.37 ± 1.8	0.14	-1.49	0.21	-1.27	0.73	-0.35
ESS	6.3 ± 4.35	6.95 ± 5.25	5.58 ± 3.31	0.84	0.2	0.36	0.92	0.59	0.54
GDS	1.9 ± 2.22	1.85 ± 2	0.95 ± 1.58	0.13	1.5	0.81	0.25	0.86	0.18
LNS	10.75 ± 1.97	10.15 ± 2.74	11.37 ± 2.29	0.47	-0.73	0.23	-1.2	0.14	-1.46
REM	3.45 ± 1.99	3.6 ± 2.21	2.76 ± 1.66	0.35	0.95	0.89	0.14	0.3	1.04
SCOPA-AUT	9.65 ± 6.77	10.5 ± 5.72	6.28 ± 3.84	0.09	1.7	0.34	0.95	**0**.**012**	2.51
SFT	50.35 ± 11.52	50.55 ± 10.77	48.16 ± 9.48	0.52	0.65	0.91	0.12	0.52	0.65
SFT-animal	23.25 ± 4.98	22.95 ± 6.03	20.89 ± 4.65	0.14	1.52	0.82	-0.23	0.39	1.06
SFT-fruit	13.8 ± 4.10	13.45 ± 3.86	14.11 ± 3.77	0.81	-0.24	0.67	-0.43	0.58	-0.55
SFT-vegetable	13.3 ± 4.66	14.15 ± 3.84	13.16 ± 3.88	0.92	0.1	0.3	1.07	0.38	0.88
STAI-state sub score	32.8 ± 8.76	31.75 ± 8.24	30.05 ± 8.19	0.34	0.96	0.65	-0.46	0.50	0.68
STAI-trait sub score	31.8 ± 8.48	31.3 ± 7.48	29.74 ± 8.93	0.38	0.89	0.92	-0.10	0.43	0.79
STAI total	64.6 ± 16.67	63.05 ± 14.7	59.79 ± 15.48	0.36	0.93	0.58	0.57	0.4	0.84
SDM	41.25 ± 9.44	38.2 ± 9.40	48.16 ± 10.12	**0**.**03**	-2.21	0.11	1.7	**0**.**003**	-3.19
MoCA	27.25 ± 2.34	27.4 ± 2.56	28.21 ± 1.13	0.3	-1.04	0.69	-0.4	0.61	-0.52
UPSIT	20.9 ± 8.79	–	33.58 ± 4.57	**<0**.**001**	-5.61	−	−	−	−

LEDD, levodopa-equivalent daily dose; H and Y, hoehn and yahr staging; ADL, ability of daily living; UPDRS, Movement Disorder Society Unified Parkinson Disease Rating Scale; BJLOT, Benton Judgment of Line Orientation Score; LNS, Letter Number Sequencing Score; REM, Rapid Eye Movement; SCOPA-AUT, Scales for Outcomes in Parkinson’s Disease-Autonomic dysfunction; VLT, verbal learning test; SFT, Semantic Fluency Total Score; SDM, Symbol Digit Modalities Score; MoCA, Montreal Cognitive Assessment; GDS, geriatric depression scale score; STAI, State-Trait Anxiety Inventory; ESS, epworth sleepiness scale score; UPSIT, University of Pennsylvania Smell Identification Test. P-values of multiple comparison were correction with Bonferroni correction. The p-values which were smaller than 0.05 were written with bold.

### 3.2. ICA components and RSN

**FIGURE 1:**
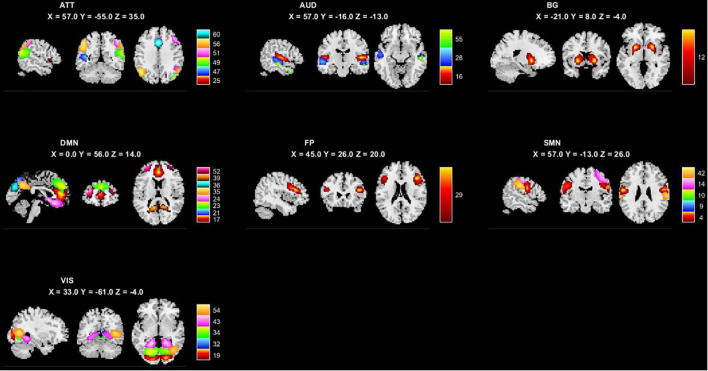
The 29 independent components and 7 networks identified by group ICA. Seven functional networks and 29 selected independent components are shown: ATT, attention network; AUD, auditory network; BG, basal ganglia network; DMN, default modal network; FP, frontoparietal network; SMN, sensorimotor network.

The minimum description length approach estimated an average of 47 components. Sixty independent components were selected for greater accuracy. Twenty-nine components were identified as meaningful according to the selection criteria described in (Section “2.5. ICA analysis”), and the selected components were grouped into seven resting-state intrinsic networks. The selected components and networks are shown in Figure 1.

### 3.3. Static FC analysis results

**FIGURE 2:**
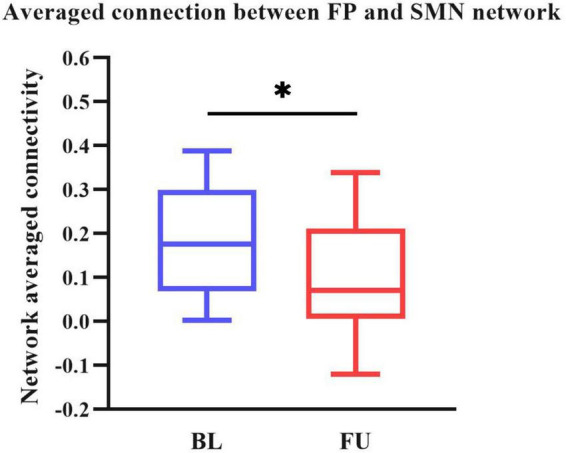
Averaged connection between FP and SMN network of PD-FU was lower than PD-BL. The *p*-value of comparison corrected using FDR correction (*: *p* **<** 0.05; *t* = **−**3.64). PD, Parkinson’s disease; BL, baseline; FU, follow-up; FP, frontal parietal network; SMN, sensorimotor network.

We performed two steps analysis to reveal static FC changes of PD in different stages. Static FC differences between the HC and PD-BL group were calculated with age, sex and education years as covariates, using two sample *t*-test. The difference between HC and PD-BL did not survive the FDR correction, which was used for multiple *p*-value correction. Differences between PD-BL and PD-FU were calculated using paired *t*-test, with FDR correction method to correct *p*-values. Results showed that network averaged connections between FP and SMN of PD-FU were lower than PD-BL (*p* < 0.05, *t* = −3.64) (Figure 2).

### 3.4. Dynamic FC analysis

**FIGURE 3:**
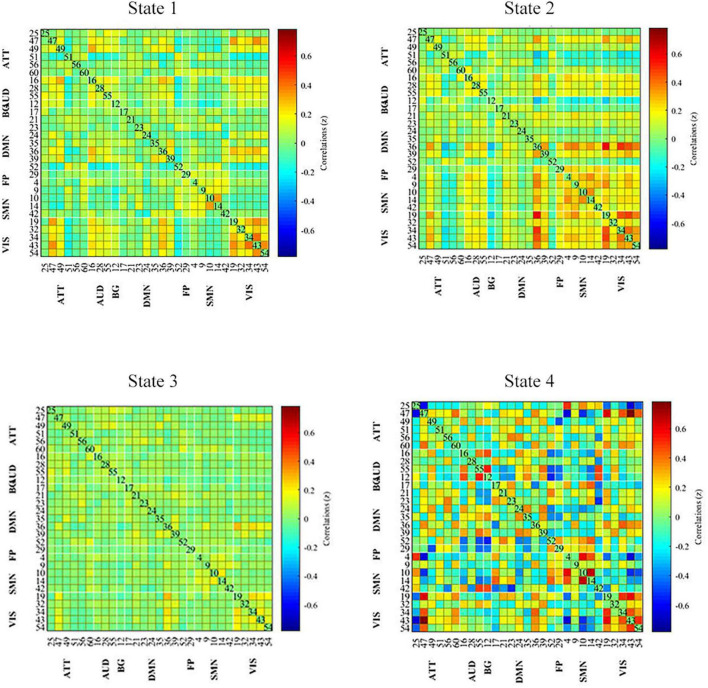
Dynamic functional connectivity state results. The dynamic FC matrices were clustered into 4 states. Averaged across subjects-specific median cluster of all participants were showed. Total occurrences for State 1-4 were 19, 22, 56, and 3%, respectively. ATT, attention network; AUD, auditory network; BG, basal ganglia network; DMN, default modal network; FP, frontoparietal network; SMN, sensorimotor network.

All dynamic FC matrices are clustered into four states according to the elbow method. 19% of the matrices clustered into state 1, characterizing positive coupling within the VIS network. State 2 contained 22% of the matrices, which characterized positive coupling within SMN, within VIS, between SMN and VIS, between DMN and SMN, and between DMN and VIS. State 3 had 56% of the matrices, showing hypo-coupling within and between all networks. State 4 was the smallest, with only 3% of matrices (Figure 3).

**FIGURE 4:**
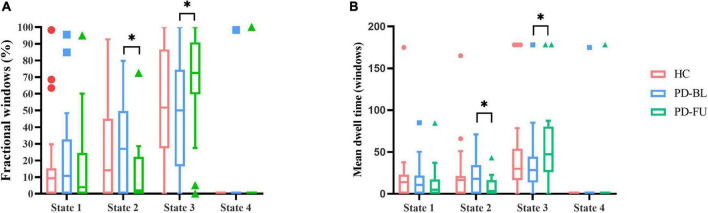
Results of temporal properties of dynamic functional state analysis. The median of fractional windows **(A)** and mean dwell time **(B)** of all subjects in each state are shown using a Tukey box plot. *: *p* < 0.05. PD, Parkinson’s disease; BL, baseline; FU, follow-up; HC, healthy control.

Temporal characteristics such as fractional windows, mean dwell time and transition number of HC group and PD subgroups were calculated. Age, sex, and education years may affect cognitive function. The effect of age, sex and education years were controlled when calculating difference between HC and PD-BL group. Results showed that there was no statistical difference between HC and PD-BL group of above temporal characteristics. Changes between PD-BL and PD-FU were performed sign rank test, because data wasn’t normality distributed. The temporal properties analysis showed that the fractional windows and mean dwell time of PD-FU at state 2 were statistically lower than PD-BL. Fractional windows and mean dwell time of PD-FU at state 3 were statistically higher than PD-BL (Figure 4).

### 3.5. Correlation analysis

**FIGURE 5:**
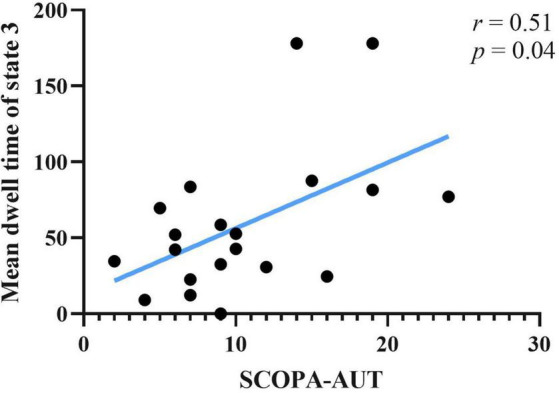
Correlation between SCOPA-AUT scores of PD-FU and mean dwell time of state 3. PD, Parkinson’s disease; SCOPA-AUT, Scales for Outcomes in Parkinson’s Disease-Autonomic dysfunction.

Correlations between clinical scale scores and measurements of rs-fMRI were computed. Clinical scales included UPDRS-I, UPDRS-II(ON), UPDRS-III(ON), SCOPA-AUT, and ADL. Rs-fMRI measures included static FC of brain areas, fractional window, mean dwell time and transition number. Age, sex and education years may affect correlation result of abovementioned score. The effect of age, sex, and education years were controlled as variances, using partial correlation method. Results showed that SCOPA-AUT scores positively correlated with the mean dwell time of state 3 of PD-FU (Figure 5). There was no correlation between clinical scores and temporal characteristics of PD-BL.

## 4. Discussion

The H and Y stages of the PD group increased after 3 years of follow-up in this study, while UPDRS-III scores (ON) did not change significantly after 3 years. As PD patients collected from PPMI dataset in this study accepted treatment, motor symptom changes were assessed by the UPDRS-III (ON). UPDRS-I and SCOPA-AUT scores of the PD group increased, and the SDM and ADL scores decreased after 3 years in our study. These results indicated that PD severity increased over time. The dopamine supplement treatment was collected as LEDD, which significantly increased after follow-up. Non-motor symptoms, such as autonomic dysfunction, progressed over time, even after long-term treatment in the present study. The UPDRS-III scores which were used to assess motor symptoms of PD, didn’t significantly change. Our results indicate that dopamine supplement treatment was not effective enough to relieve all non-motor symptoms in this study.

Our result showed that network averaged connection between FP and SMN of PD-FU was lower than PD-BL. FP is associated with execution function. The decreased connection between FP to SMN in PD-FU may be associated with worsen of motor symptoms. While we didn’t find statistical correlation between decrease of network connection and clinical assessment scores.

Results showed that PD-FU spent more time at state 3, which characterized as hypo-coupling between networks. PD-FU spent less time at state 2, which was characterized as positive coupling between networks. These results indicated that coupling between networks of PD-FU decreased and may converted to hypo-coupling state. The loss of coupling may associate with the worsen of PD. These results are consistent with those of Li et al. (2021), which showed that the fractional windows and mean dwell time of states that characterized positive correlations between FP and SMN decreased in PD compared to HC (Chen et al., 2021). In Li et al.’s (2021) study, the mean dwell time of the above ON state increased compared with the OFF state, indicating that dopamine depletion affects functional connectivity stability between FP and SMN (Chen et al., 2021).

Autonomic dysfunction was widespread in PD and occurred at an earlier stage. Autonomic dysfunction, such as gastrointestinal dysfunction in PD patients, was a risk factor for falling, with severe consequences (Kwon et al., 2021). The SCOPA-AUT scale was used in this study to assess autonomic dysfunctions. The autonomic functions of PD patients at FU were impaired in our study. The correlation results showed that the SCOPA-AUT scores of PD-FU positively correlated with the mean dwell time of state 3. Above results indicated that the increase of hypo-coupling state correlated with autonomic dysfunction. Our results in line with precious studies, which showed the correlation between functional connectivity of RSN and autonomic dysfunctions (Ashraf-Ganjouei et al., 2018; Dayan et al., 2018; Li et al., 2021; Nakano et al., 2021).

## 5. Limitations

There are some limitations to this work. Only 20 PD and 19 HC patients were collected from PPMI in this study. PD patients were only followed up for 3 years, which was much less than the entire length of PD duration. A larger scale and longer visiting period for the longitudinal study is needed in future studies. Most of the PD patients in the recent study received treatment, and the UPDRS-III scores of the OFF states were missing. PD patients did not undergo MRI scans before accepting any treatment. We cannot entirely remove the treatment effect. Although some studies revealed that dopamine treatment rarely affected non-motor symptoms, randomized controlled trials are needed in the future.

## 6. Conclusion

We found four distinct dynamic FC states in PD patients according to dynamic functional correlations within and between RSN. The state 2 of our study showed positive coupling within and between SMN and visual network, while the state 3 showed hypo-coupling through all RSN networks. Our finding indicated that PD-FU patients spent more time in hypo-coupling state (state 3) than PD-BL. The increase of hypo-coupling state and decrease of positive coupling state might correlate with the worsening of non-motor symptoms in PD patients. The dynamic FC analysis may be used as monitoring tool for non-motor symptoms and disease progression.

## Data availability statement

The original contributions presented in this study are included in the article/supplementary material, further inquiries can be directed to the corresponding authors.

## Ethics statement

The studies involving human participants were reviewed and approved by the all data used in this study were downloaded from PPMI dataset. The patients/participants provided their written informed consent to participate in this study.

## Author contributions

YC: methodology, investigation, formal analysis, and writing–original draft. QS and RT: methodology. SM, CL and XZ: review and editing. All authors contributed to the article and approved the submitted version.

## Footnotes

1
https://www.ppmi-info.org/access-data-specimens/download-data

2
www.ppmi-info.org

3
https://web.conn-toolbox.org/

4
https://trendscenter.org/software/gift
